# Group-based, person-centered diabetes self-management education: healthcare professionals’ implementation of new approaches

**DOI:** 10.1186/s12913-019-4183-1

**Published:** 2019-06-11

**Authors:** Vibeke Stenov, Gitte Wind, Michael Vallis, Susanne Reventlow, Nana Folmann Hempler

**Affiliations:** 1 Department of Nursing, University College Copenhagen, Copenhagen, Denmark; 2 Dalhousie University, Halifax, Nova Scotia Canada; 3 Department of Public Health, University of Copenhagen, Copenhagen, Denmark; 4 Diabetes Management research, Health Promotion, Steno Diabetes Center Copenhagen, Niels Steensens Vej 2, DK-2820 Gentofte, Denmark

## Abstract

**Background:**

Healthcare professionals’ person-centered communication skills are pivotal for delivering successful diabetes education. Many healthcare professionals favor person-centeredness as a concept, but implementation in practice remains challenging. Today, programs have often a fixed curriculum dominated by biomedical issues. Most person-centered methods are developed targeting individual consultations, although group-based programs are a widespread and efficient method of support. Person-centeredness in group-based programs requires a change in practice towards addressing biopsychosocial issues and facilitating group processes. The objective of this study was to explore how healthcare professionals implement new approaches to facilitate group-based, person-centered diabetes education targeting people with type 2 diabetes.

**Methods:**

The study was guided by action research and divided into three studies: investigation, development, and pilot using a variety of qualitative methods. In the first study; observations across five settings were conducted. Forty-nine group participants and 13 professionals took part; the focus was to investigate approaches that supported or hindered person-centeredness in groups. Observations were supplemented by interviews (*n* = 12) and two focus groups (*n* = 16) with group participants, as well as interviews (*n* = 5) with professionals. In the second study; 14 professionals collaborated in two workshops to develop new approaches. In the third study, new approaches were pilot-tested using observations in three settings. Twenty-five group participants and five professionals took part. The analysis of the pilot test led to the final workshop where six professionals took part.

**Results:**

Implementation was characterized by three categories. Some professionals chose not to implement the methods because they conflicted with their practice relying on the biomedical model. Other incorporated some approaches but was unable to structure the process, leaving participants uncertain about the aim. Finally, one setting succeeded with implementation, tailoring content and processes to group participants’ needs.

**Conclusion:**

The use of action research created context-sensitive approaches and increased professionals’ readiness to implement. More attention should be paid to systematic training of professionals. Training should be structured stepwise incorporating techniques directed towards existing skills including ample time to train and reiterate skills.

**Electronic supplementary material:**

The online version of this article (10.1186/s12913-019-4183-1) contains supplementary material, which is available to authorized users.

## Background

Group-based, person-centered diabetes self-management education is offered widely and increasingly associated with benefits that include higher patient satisfaction, improved health outcomes, peer support, and increased cost effectiveness [1–6]. Research has generally focused on outcomes of group-based diabetes programs and less on form and content. However, group-based diabetes education is highly complex and challenging for healthcare professionals (HCPs) to facilitate due to variations in intended purpose, content, and format [7, 8]. Many group-based programs described in the literature do not clearly identify or describe the communication skills practiced by HCPs to support self-management. In fact, the majority of studies that report successful outcomes in group programs do not include a detailed description of specific communication skills used by facilitators [9].

Some studies have identified facilitators’ communication skills as more important than their professional backgrounds [10]. In particular, professional skills related to problem solving, goal setting, and facilitating active participation and group dynamics have been identified as key factors in supporting health behavior changes among group participants [3, 7, 11–13]. These skills support a person-centered approach, which is currently the nest evidence in diabetes self-management education and of prime importance to good clinical practice [14, 15]. In patient education, the concept of person-centeredness describes a shift away from one-way transmission of content from medical experts to passive listeners and toward actively incorporating participants’ experiences, concerns, and needs into the curriculum [16]. Thus, the person-centered approach promotes a collaborative process in which the role of the HCPs is to guide progress, catalyze motivation, and provide the right amount of information at the right time to encourage learning among group members [16–18]. Yet many HCPs have not received training in how to switch practice from “teach and tell” to collaborate and empower [18].

Results from the second Diabetes, Attitudes, Wishes and Needs study (DAWN2) revealed that HCPs experienced substantial barriers to providing person-centered diabetes self-management education [19]. Thus, there is still a missing link in the process of translating person-centered research approaches into the implementation of skills in clinical practice. Further, most HCPs have not been trained in running groups in a manner other than the didactic lecture [16]. To the best of our knowledge, no studies have explored whether and how HCPs who have received training in effectively facilitating group-based, person-centered diabetes education implement the new approaches in practice. Therefore, the aim of this article is to explore how HCPs implement new approaches to facilitating group-based, person-centered diabetes self-management education after professional development activities.

## Methods

### Design

The study design was guided by action research [20], which is suited to simultaneously integrating research into practice and supporting change [21]. An action research approach allows practitioners to collaborate in the process of creating research knowledge, similar to the key principles of dialog and active involvement in person-centered education [21]. By using action research, we strived to empower HCPs to be highly engaged in both the development of the research and the subsequent integration of activities into practice.

### Setting and purposeful sampling of the study

**Fig. 1:**
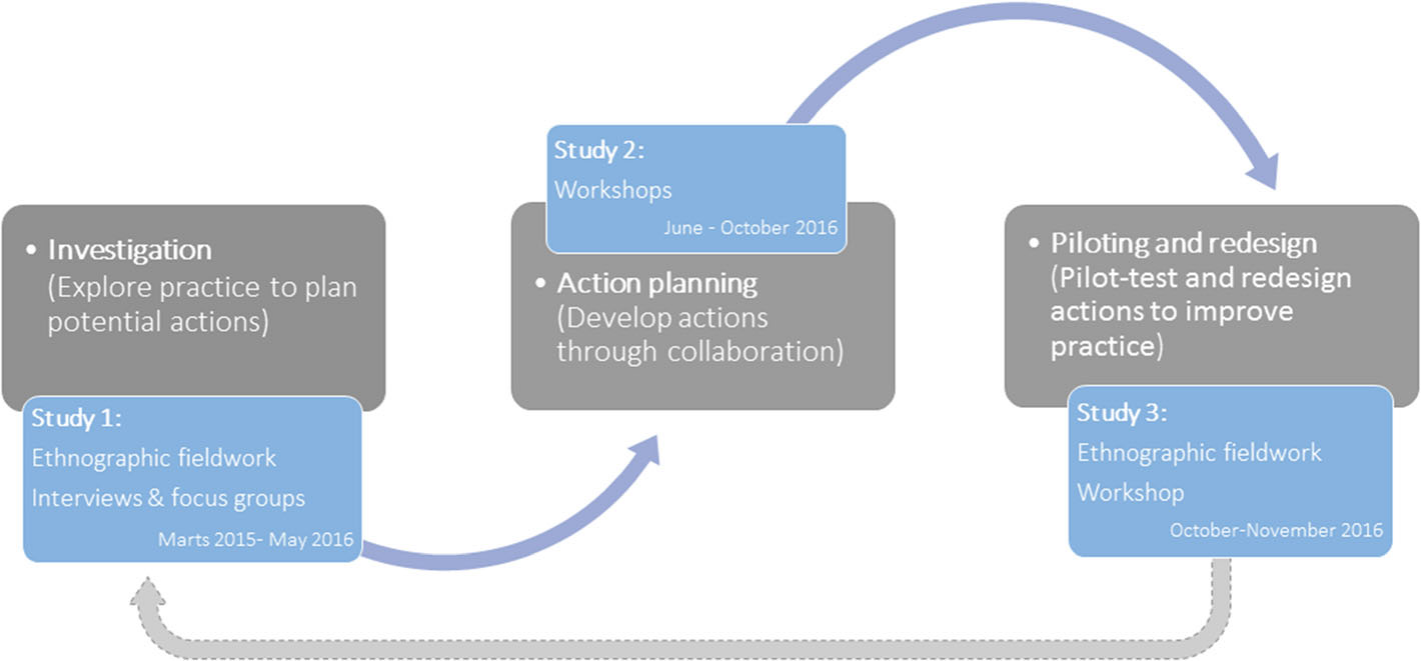
Model of the study design inspired by action research

Qualitative methods such as fieldwork, interviews, focus groups, and workshops were used to collect data for the identification and selection of information-rich cases [22, 23]. The model of study design inspired by action research is illustrated in Fig. 1.

Eight hospital and municipal settings in the greater area of Copenhagen were initially contacted. The settings varied in relation to geographical location in the region and size; yet representative in terms of scope and content. Additionally, the settings were chosen to obtain participants who varied in terms of profession, level of postgraduate training, and experience. One hospital and four municipalities agreed to participate. Thus, we collaborated with four registered nurses, four physiotherapists, five dietitians, and an occupational therapist across five settings (Table 1). In addition, researchers with public health, communication, and psychology backgrounds participated in the study.

**Table 1:**
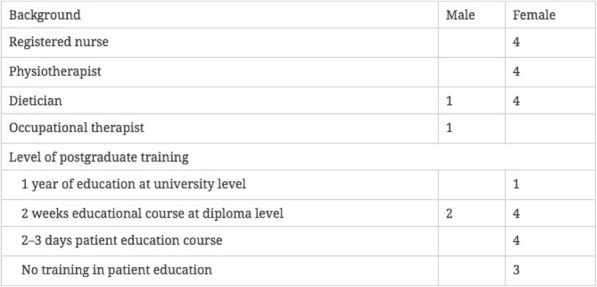
Characteristics of HCPs participating in the study

HCPs in the settings were selected using purposeful sampling based on availability and genuine willingness to participate in the study, attend in discussions, and were interested in developing professional skills [24]. Participation required: 1) access to conduct ethnographic fieldwork in their practice; 2) participation in three professional workshops; 3) individual meetings to customize group-based, person-centered approaches to match local circumstances, existing skills, and perceived needs before pilot testing; and 4) willingness to pilot test group-based, person-centered approaches in practice.

### Data content and data collection

Data collection included three sub-studies, and each of these was followed by an integrated analysis of new and previously collected data to develop and plan the next phase.

### Study 1: investigation

In the first study phase, field observations were used to understand local context and HCP baseline skills, which took place from March 2015 to May 2016. Subsequently, we conducted semi-structured interviews with HCPs (*n* = 5) from the five settings (one hospital and four municipalities) and with group participants (*n* = 12) from three settings; we conducted two focus groups with participants (*n* = 10, *n* = 6) at the remaining settings. Interviews and group discussions focused on HCP and participant experiences of setting-specific approaches. The fieldwork and interviews were essential for planning the professional development process and is reported elsewhere [25].

### Study 2: action planning

**Fig. 2:**
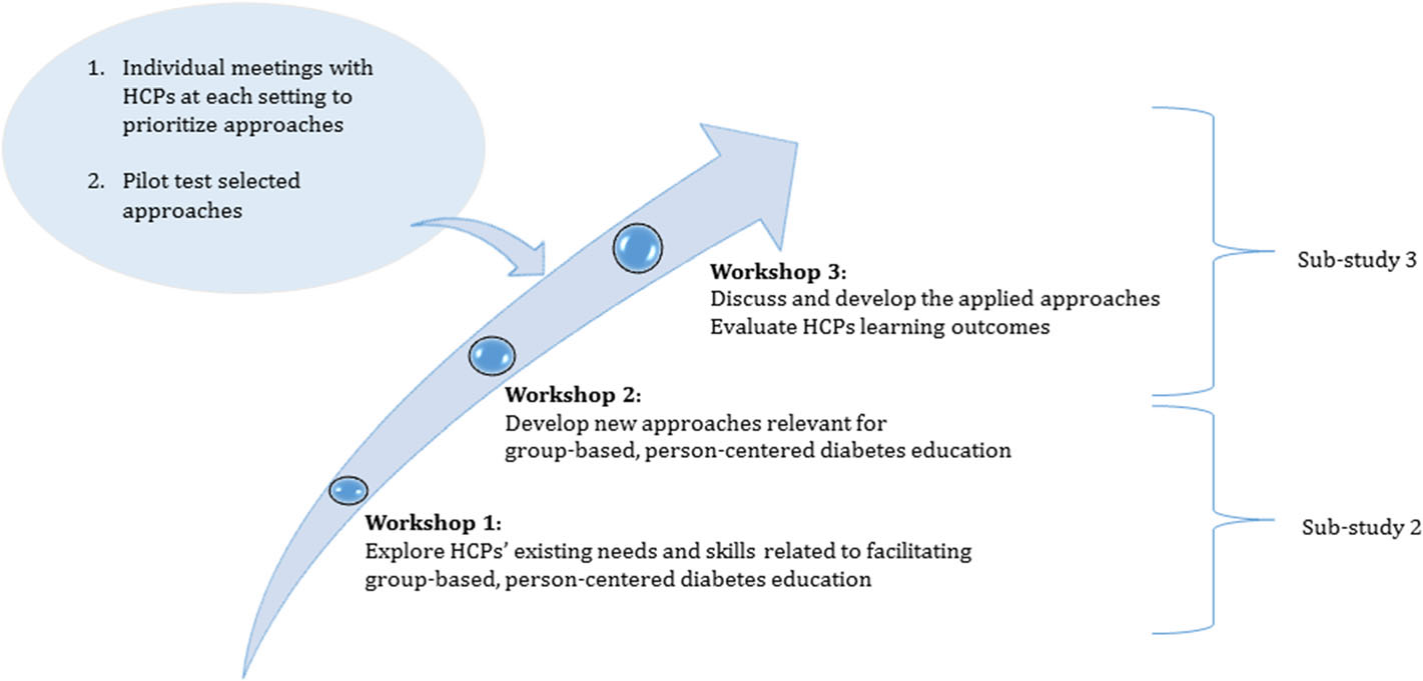
Overview of the intervention

In the second phase of the study, findings from the first phase were used to plan professional development workshops. HCPs (*n* = 14) from the five observed settings collaborated in two workshops from June to October of 2016. We used the term workshop to emphasize the user-driven and collaborative research approach. The overall aim of the workshops was to develop, in collaboration with HCPs, new approaches supporting the implementation of new strategies to facilitate group-based, person-centered diabetes self-management education. Each workshop focused on a single goal in pursuit of the overall aim. Figure 2 depicts an overview of the intervention.

Each workshop lasted 3 h and invoked a series of exercises to promote participation, reflection, and dialog. ‘The Health Education Juggler’ [26] and the principles of Motivational Interviewing in Groups [27] were presented and used to delineate and evaluate essential elements of facilitating high-quality group-based, person-centered diabetes education. The Health Education Juggler model comprises of four equally important educator roles: Embracer, Facilitator, Translator, and Initiator [26]. The Embracer is empathetic and intuitive. The Facilitator enables reflections on limitations and challenges in everyday life. The Translator conveys disease-specific knowledge in an understandable and implementable way. The Initiator creates motivation for behavior change [26]. Juggling is a metaphor for HCPs who must simultaneously manage, master, and switch between these roles when facilitating group-based, person-centered diabetes education [26]. Principles underpinning MI in groups [27] were used to support HCPs in the understanding of group participants’ needs, preferences, and values and to tailor the educational approach to address them. Furthermore, to facilitate dialog within the group, and to emphasize participation and collaboration [27]. MI in groups were useful to prompt open-ended questioning, minimizing statements and avoiding argument, promoting unconditional acceptance by demonstrating non-judgmental curiosity, and to facilitate individual reflections. Thus, the focus was not to persuade group participants to behavior change, but to facilitate active engagement and promoting person-centered care.

The Health Education Juggler’ [28] and MI techniques [27] inspired to a self-reflection tool, aiming at stimulating HCPs’ self-reflection about their professional skills by identifying their strengths and areas in need. The development of the self-assessment tool is presented elsewhere [29]. Furthermore, the model was used to evaluate HCPs’ implementation of new approaches.

The workshops were semi-structured in the sense that the research group facilitated the process to maintain a focus on topics related to incorporating new approaches into practice. More specifically, the workshops and collaboration with HCPs were planned and conducted by the first author in close collaboration with a researcher experienced in user-driven innovation. In addition, a research team consisting of a researcher, a research assistant, and a student assistant participated in the workshops. The researchers’ role was to facilitate workshop processes to investigate HCPs’ experience, preferences, and needs for developing professional skills and to present and discuss potential group-based, person-centered approaches. All workshop processes had two purposes: to collect data and to explore potentials of the new approaches to inspire and assist HCPs to facilitate group-based, person-centered diabetes education. Workshops included a variety of methods, such as reflection sheets, case scenarios, dialog tools, and video clips, aiming a promoting dialog and facilitate the process. These methods allowed HCPs to generate their own ideas and discuss them. Insights from the workshops enabled the researchers to refine the prototypes. Table 2 summarizes the workshop activities.

**Table 2:**
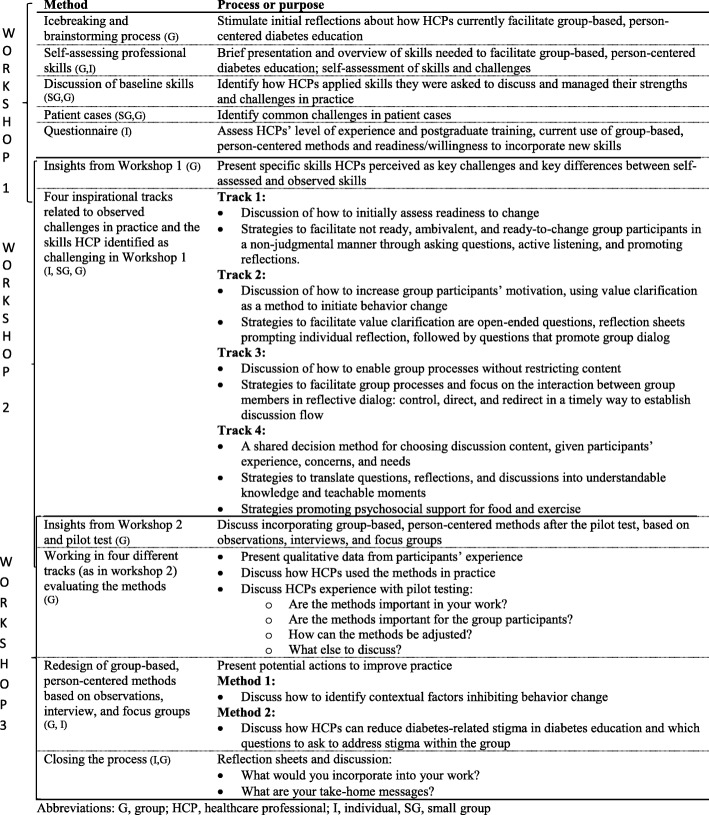
Overview of workshop activities in workshops

*Abbreviations*: *G* group, *HCP* healthcare professional, *I* individual, *SG* small group

Relevant theoretical models provided inspiration for techniques and tools supporting implementation of new approaches: principles of person-centered communication [16], motivational interviewing [27, 28], and readiness assessment [30]; problem-solving and goal-setting techniques grounded in social cognitive theory [31]; emotional-behavioral strategies [32]; and group facilitation skills [33].

### Study 3: piloting and redesign

In the third phase of the study, findings from fieldwork and analysis of the two workshops were used to plan individual meetings with HCPs in which techniques and tools appropriate for each setting were discussed, further developed, and selected. Thus, the length of the program was individually planned with each setting due to logistically reasons and local resources. However, we highly prioritized individual meetings with all HCPs in which all approaches were iteratively developed in collaboration with HCPs throughout the intervention. Considerable effort was done to develop and remediate techniques and tools to make the facilitation of person-centered methods as confident, comfortable, and feasible depending on HCPs’ existing professional skills, experiences, level of postgraduate training, and local resources.

**Fig. 3:**
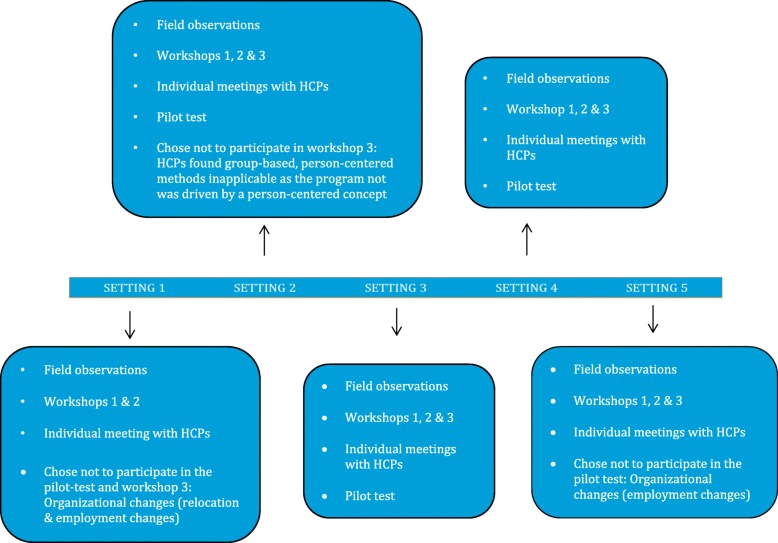
Collaboration with study settings

We then pilot tested how HCPs incorporated selected approaches in three settings that included 25 individuals with type 2 diabetes and five HCPs. Techniques and tools tested in the settings are illustrated in Additional files 1, 2, 3 and 4 (presentation exercise, tool to assess readiness to change and facilitation techniques, tool to facilitate reflection and dialog about exercise habits, tool to facilitate reflection and dialog about eating habits). Field observations were conducted between August 2016 and November 2016. Two settings dropped out prior to the pilot phase due to issues associated with organizational resources such as relocation and employment changes. Figure 3 details the collaboration process at each setting.

The analysis of the pilot test results led to the final workshop at which we collaborated with HCPs (*n* = 6) to develop new actions to improve practice, discussed how to improve techniques and tools, and evaluated the process and the HCPs’ learning outcomes.

### Data analysis

Data comprised interview and workshop transcripts, field notes from the pilot test, and program documents such as PowerPoints, program schedules, and e-mails; all were analyzed using systematic text condensation [34]. Initially, data were read closely to obtain an impression of the whole. Second, meaning units were identified, exploring HCP facilitation of group-based, person-centered diabetes self-management education. Thirdly, meaning units were sorted into three categories about how HCPs implement new approaches after professional development, and then they were condensed. Finally, the content of the categories was summarized into more general descriptions of themes.

## Results

Three core categories were identified, describing how HCPs implemented new approaches after participating in professional development about facilitation of group-based, person-centered diabetes self-management education.

### Increased awareness but implementation challenges remain

HCPs from all three settings were very engaged in the workshops. They considered themselves highly ready to change, and they agreed with the theoretical principles related to facilitating group-based, person-centered diabetes self-management education. Additionally, they were engaged in self-assessing strengths and areas in need of professional development. They easily identified common challenges described in patient cases representing typical scenarios observed in practice and were engaged in developing specific tools and techniques to enable practical application.

Participating in professional development promoted an increased awareness of the person-centered approach. However, some inconsistency emerged when it came to actual implementation. The shift away from being an expert who defined the content and provided recommendations (teach and tell) to include person-centered skills was particularly challenging. As one educator reported, “I miss a chance to give them input and concrete knowledge—the participant has to talk all the time, but I really need to tell them something concrete.” (HCP, setting 1).

The organizational capability for implementing person-centered approaches was highly variable. In general, organizational changes, such as new locations or jobs, were common reasons for cancellations in the pilot phase: “We just moved and it didn’t really go as planned so I have to cancel tomorrow.” (E-mail setting 1).

Although one setting agreed to pilot test some approaches, it was important for HCPs in this setting to select approaches that could easily be applied within an existing structured curriculum informed by a clinical agenda. The following excerpt from field notes describes how HCPs in this setting applied tools as an add-on method to the established curriculum. The HCPs used techniques to elicit group participants’ preferences but did not subsequently align the curriculum to the group participants’ preferences and current circumstances:

The first HCP expresses: “The time is very limited but today we have a guest (refers to the researcher), so we really want to thank you for your help and collaboration on this.” At the same time, the second HCP stands ready in front of the PowerPoint presentation with the headline “Diabetes and Diet”, looking at his watch to indicate that this is taking too much time. The first HCP continues, “Now we will ask you to fill in these sheets.” The HCP explains that the hand-out contains questions asking the group participants to reflect individually on what to change and their readiness for change. The first HCP explains further: “Then we will collect your answers and give it back to you at the end of the program (5 weeks later) to see whether the education has given you further motivation.” (Pilot test, setting 1)

The knowledge gained from group participants was not used to make the content meaningful; instead, the HCPs from this setting were part of a cultural milieu more akin to paternalism, in which behavior change was believed to result only from increased knowledge. The need to apply person-centered approaches was not fostered by a supportive organizational culture or perceived as particularly meaningful. However, the HCPs from this setting gained an important understanding from participating in the workshop; they realized that their program was not driven by person-centeredness. They consequently found the methods not applicable in practice. The HCPs became increasingly skeptical and, after the pilot test, decided that the new approaches were less important than the long-established topics in their curriculum:

My colleague and I had a meeting yesterday. We came to the conclusion that we have different views on the program. We think of our program as information and sharing of knowledge, but your view is more on the changing process. We think that this process comes after they know more about their disease. Therefore, it is difficult for us to implement what we have learned from you. (E-mail, setting 2)

### Readiness to change but unable to facilitate and create clearness

HCPs from another setting were ready and motivated to learn new approaches and apply them in practice. As one stated: “To become an educator, it never ends. It requires constant development and you have to be mindful about it when you begin as an educator and be open to it.” (HCP, setting 5).

After professional development, HCPs from this setting had an increasing focus on using participatory learning techniques to actively involve the whole group. They used open-ended questions to engage group participants in reflection. Additionally, they allowed group discussions during which participants shared their experiences, needs, and concerns. However, in the effort to avoid the medical model, structure fell by the wayside. The HCPs moved so far away from the expert role that taking control of the process when needed was challenging. One educator stated the following about this way of teaching:

The last sessions were really challenging because we tried to throw away the structure, then I thought—do we really know what we are doing? It really required personal capacity, it required energy, and I felt really exhausted, right! I thought along the way, are we where we should be, and are we at all achieving the content? (HCP, setting 3)

Many HCPs planned exercises that included personal reflections. However, there was often a lack of transparency about the process, and HCPs frequently jumped into activities without explaining the aim. The structure occasionally became undefined and unproductive, as illustrated in a dialog between HCPs in the final workshop:

HCP 1: It’s more about feelings and personality and that kind of stuff. We want them to reflect, but there is not really a professional content to disseminate. We just tried to be in the room and let the dialog flow, instead of control. We were just floating with the dialog and then followed where it went.

HCP 2: I once had a man who said I don’t get anything out of it; it’s only chitchat.

HCP 3: It’s funny, because it’s on the other end, right! We have made up a slideshow and decided then we do this, and questions—it’s kind of annoying. And the opposite end, when we get completely out on a sidetrack and we never end up discussing what we planned. (HCPs, setting 3 & 5)

In the effort to abandon the role as the expert who defined the content, HCPs tended to adopt a narrower focus on goal-oriented concepts. For example, they used tools and techniques focusing on goal setting and action planning. In particular, they tended to force some group participants who were not ready to change to set goals that they had not created themselves, as one HCP articulated:

He wasn’t really interested in changing anything. I really thought it was difficult not to put the words into his mouth. I asked him what he wanted to change or simply just try out the next week. He just said, I can try if you want. Then I said you shouldn’t do it for me; it’s for your own sake. After the program he walked directly into another room and said, now they [HCPs] want me to lose weight. (HCP, setting 5)

### Content and process tailored to the needs of group participants

In one setting, a HCP collaborated with group participants, working from their agenda and tailoring the content to their expectations, needs, and concerns. The HCP mastered the complexity of balancing content and process skills within the program and used didactic theory to expand and consolidate discussions directed towards learners’ perceived needs. As the HCP expressed:

It’s important to begin with the participants’ needs and then facilitate behavior change from that point of view. Then, supporting them to be clearer about how they will work on it in their own way. You don’t facilitate that by traditional didactic teaching, because then you don’t know what the participants want. (HCP, setting 4)

The HCP was particularly concerned about group processes in terms of creating a positive group climate and was able to both maintain a focus on exploring important issues and simultaneously move the process forward. In the following quote, the HCP describes how she intentionally handled individual group members with potentially disruptive behavior by using acknowledging responses to avoid sidetracking the dialog:

I’m now better at directing the group. I was really well prepared to handle one participant, and I talked with my colleagues about how to handle her. In general, stopping people without making the atmosphere unpleasant is difficult but very important. I put lot of effort into telling them initially my expectations and the importance of making room for everyone. Maybe that is why the participants don’t think it’s awkward (HCP, setting 4).

The HCP used strategies to both accelerate and slow the pace of discussions. When she slowed the pace, she used group discussion methods to help participants interact with each other while they explored concerns and enabled the whole group to be empathic and collaborative in offering suggestions. The learning experience enriched the whole group. The HCP described how she facilitated the group dialog and acknowledged the challenges of behavior change:

One participant had an issue that I’m quite sure everyone in the group had in mind. Then we talked a lot about that issue, because it’s something about how to stick to new habits, right. It’s quite difficult for everyone. I actually thought we talked about it in a way without blame and shame; we talked about it in a constructive manner. Has anyone in the group tried it and has any ideas or solutions? (HCP, setting 4)

Finally, the HCP used participatory learning strategies and mastered simultaneous interventions on both group and individual levels by making space for self-reflection and group discussion of issues. Thus, she was able to structure the program on both educational objectives and individual needs:

It’s useless for the participants that we teach and tell them about internal and external motivation. It’s much more important that they get the chance to articulate by themselves what is motivation. Then give them a chance to mirror their different ideas. They don’t achieve that from a lecture about motivation. They have to be actively involved. (HCP, setting 4)

## Discussion

We found that HCPs applied new approaches quite differently after participating in professional development about group-based, person-centered diabetes self-management education. In general, there was a broad consensus in support of the concept, HCPs expressing readiness to change. However, the actual implementation was challenging with many HCPs experiencing barriers. Barriers to implementation included existing frameworks in which HCPs were experts who disseminated content, and also issues associated with organizational resources. It appears common that HCPs had a genuine readiness to leave their roles as experts until they began to implement it. Implementation precipitated a swing to the opposite pole, which resulted in unstructured processes and an inability to direct group discussions when needed. Further, one HCP expressed dissatisfaction with not being able to maintain the expert “teach and tell” style. However, one HCP mastered the complexity of balancing process and content, facilitating person-centered processes and simultaneously providing expert knowledge in manageable amounts at the right time. It appears that uptake of this model of care is currently dependent on the individual characteristic of the HCP. This raises the question whether HCPs, not naturally inclined to this model, can learn to stop doing what comes naturally and adapt this new style. Operationalizing the criteria to define competency in delivery of person-centered care will allow for this question to be evaluated.

### Balancing paternalistic and consumerist extremes

The implementation of new approaches was characterized by two extremes; either HCPs took on the role of an expert by defining the content and providing recommendations following a fixed curriculum or, in the effort to abandon unwanted paternalism, tended to swing so far away from the expert role that the group had all the control. This is consistent with Cribb and Entwistle, who argue that current perceptions about shared-decision approaches tend to be interpreted too narrowly in application [35]. This reveals a frequent misconception of the HCP role at the extremes of either paternalism or consumerism [35]. They argue for a broader middle path between paternalistic and consumerist models that seeks to work with the autonomy and responsibility of both HCP and patient [35]. However, Cribb and Entwistle also question whether it is reasonable to expect that all HCPs have the knowledge and skills to navigate this comprehensive middle path and whether it merely represents an ideal that is difficult to implement in practice because it is so far from current clinical norms [35].

### The complexity of implementing new approaches

The complexity of applying new approaches in practice is further supported in a study by Lim and Morris, which estimates that only 10% of learning actually transfers directly to performance [36]. The professional development conducted in this study emphasized personal agency, primarily using HCPs as change agents, and focused less on how organizational and structural factors influence the application of new approaches in practice. Nevertheless, we found that a lack of organizational resources was a fundamental cause of the inability to apply new approaches despite the fact that all HCPs reported being highly ready to change. Including organizational and structural factors in change implementation strategies were found to be critically important and require support at all levels, e.g., a supportive organizational culture and increased leadership support [37]. Weiner suggests that assessing readiness to change among HCPs is an important and necessary step but argues for the need to also assess organizational readiness to change, in which the entire organization expresses a collective commitment to the changes required to successfully apply new approaches [38].

### Successful components in the present study design

When examined in light of theories about key features of new approaches that support their implementation, the professional development described here paid careful initial attention to the current local contexts in which the intervention took place by observing and identifying potential enablers and barriers [25]. We also identified a common ground in the form of agreement among HCPs on the core principles of delivering group-based, person-centered diabetes self-management education [29] and high readiness to change, implying that HCPs were committed to take action. We engaged HCPs as change agents and customized approaches to match their local circumstances, perceived needs, and existing skills. Finally, we collaborated closely with HCPs in the design of concrete, context-specific techniques and tools to enable practical application [18, 39].

### The benefit of designing a longitudinal study

Although participatory strategies were primarily used to change practice in our study, it could have been beneficial to provide additional interventions at the level of HCPs. Evidence suggests that changing professional skills requires systematic training that includes ample time to rehearse and reiterate skills, mediated by supervisors who are able to facilitate experiential learning [18]. Detailed and descriptive video feedback is also beneficial to developing professional skills. In particular, learning by experimentation that is facilitated in an active small group environment has been shown to strengthen peer support and mutual learning [18].

However, this study had limitations related to its time frame. It was designed and planned to occur over a relatively short period of time for logistical reasons, despite the fact that evidence highlights the importance of a more flexible and incremental approach used within a longitudinal design. In addition, professional development program design should allow ample time for robust communication, increasing complexity, and adjustment to HCPs’ needs and preferences.

### Tools are not enough to promote person-centered care

In this study, we chose to include several different tools and techniques drawing on a person-centered approach. However, a study by Lloyd et al. [40] argues that tools and techniques to perform group-based, person-centered diabetes education are not enough to facilitate person-centeredness when they are used in isolation. Developing HCPs’ professional communication skills and fundamental mindset based on a person-centered approach is crucial and must be accomplished before bringing specific techniques and tools into play [40]. This is consistent with a review by Fisher et al. [41] who describe two crucial steps that HCPs, to enhance an encourage improvements in self-management, must complete before tools are brought into play. First, they must be supported in shifting their mindset from a traditional hierarchical approach to a collaborative and empathic approach and they must move from a traditional educational approach of delivering information toward listening to address motivational needs and obstacles among people with T2DM. The second and equally fundamental step is to support HCPs in applying empathic relationship-building strategies [41]. Thus, empowering HCPs to master the professional skills required to facilitate group-based, person-centered diabetes education may be a long process, because it requires knowledge about theoretical paradigms, conscious self-reflection, participation in learner-centered and practice-oriented training programs, and, importantly, continuous training to maintain skills over time [42]. In particular, interactive skills-training workshops, such as role play-based training that emphasizes practical skills, has been found to improve professional skills in comparison to theory-heavy presentations [43]. Thus, developing HCPs’ professional communication skills and fundamental mindset based on a person-centered approach is crucial and must be accomplished before bringing specific techniques and tools into play.

### Study limitations

Although participatory strategies were used in our study primarily to change practice, it could have been beneficial to provide additional interventions at the level of HCPs. Particularly, evidence suggests that changing professional skills requires systematic training that includes ample time to rehearse and reiterate skills, mediated by supervisors who can facilitate experiential learning [18].

Moreover, the professional development conducted in this study emphasized HCPs as change agents and focused less on how organizational and structural factors influence the application of new approaches in practice. Nevertheless, a lack of organizational support was fundamental for implementing new approaches, although all HCPs reported being highly ready to change. In line with organizational and structural factors, implementation strategies were found to be critically important for change and require a supportive leadership and organizational culture [37].

## Conclusion

This study shows that participatory methods in professional development can create context-sensitive methods to be implemented in group-based, person-centered diabetes education. However, more attention should be paid to supporting HCPs while implementing new methods in practice including the organizational and structural factors in the change process.

Identifying how HCPs apply person-centered approaches for facilitating group-based patient education is highly relevant for several reasons. It supports experts in professional development programs in considering possible obstacles and structuring a stepwise curriculum incorporating a variety of training techniques directed towards existing skills. It can engender more realistic expectations of outcomes of professional training, i.e., incremental learning is required to address increasing complexity. Finally, our study highlights the importance of taking essential organizational and structural factors into account before identifying potential professional development strategies.

## Additional files

Additional file 1: Presentation exercise. (PDF 130 kb)

Additional file 2: Tool to facilitate reflection and dialog about exercise habits. (PDF 112 kb)

Additional file 3 Tool to facilitate reflection and dialog about eating habits. (PDF 114 kb)

Additional file 4: Tool to assess readiness to change and facilitation techniques. (PDF 443 kb)

## Data Availability

The authors do not wish to make the data available because it contains information that could identify specific individuals.

## Abbreviations

HCP: Healthcare professional
MI: Motivational Interviewing
