# Supplementary material for: Group-based, person-centered diabetes self-management education: healthcare professionals’ implementation of new approaches
Source: BMC Health Serv Res. 2019 Jun 11;19:368. doi: 10.1186/s12913-019-4183-1 (PMC6558764; doi:10.1186/s12913-019-4183-1)
Supplement: Supplementary file 1 — Presentation exercise. (PDF 130 kb) [file 12913_2019_4183_MOESM1_ESM.pdf]

Supplementary file 1. Presentation exercise

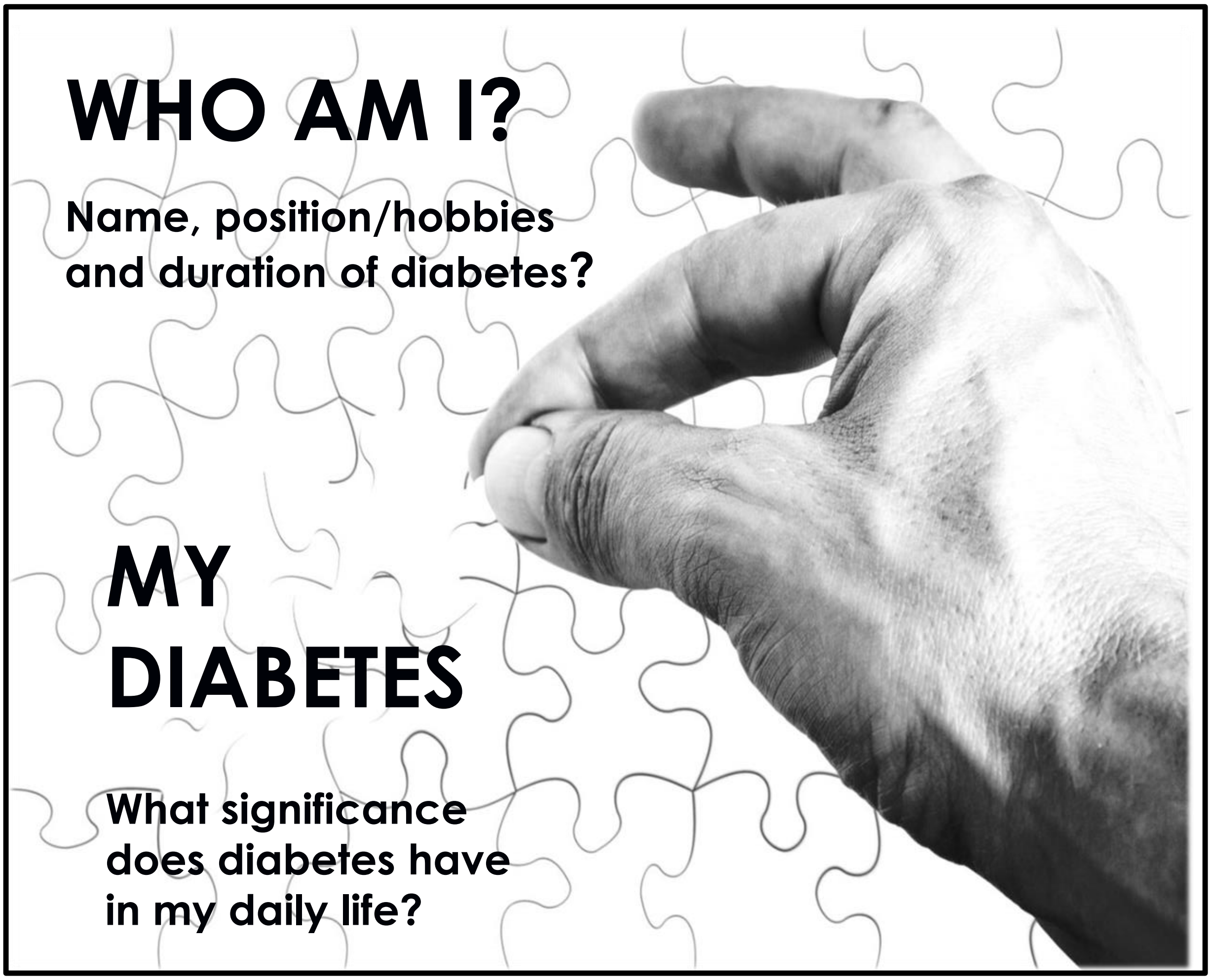

MY ANSWER
