# Supplementary material for: Group-based, person-centered diabetes self-management education: healthcare professionals’ implementation of new approaches
Source: BMC Health Serv Res. 2019 Jun 11;19:368. doi: 10.1186/s12913-019-4183-1 (PMC6558764; doi:10.1186/s12913-019-4183-1)
Supplement: Supplementary file 2 — Tool to facilitate reflection and dialog about exercise habits. (PDF 112 kb) [file 12913_2019_4183_MOESM2_ESM.pdf]

Supplementary file 2. Tool to facilitate reflection and dialog about exercise habits

MY EXERCISE HABITS

A number of situations are described below that can make it hard to stick to exercising regularly. Please circle the scale below. How strongly do you agree with the following statements?

I find it difficult to exercise…	0 (Strongly disagree)	1	2	3	4	5	6	7	8	9	10 (Strongly agree)
…when I am in a bad mood (tired, experiencing personal problems, feeling low or depressed)	0	1	2	3	4	5	6	7	8	9	10
… when it is inconvenient (poor weather, traveling, having visitors, gym is closed)	0	1	2	3	4	5	6	7	8	9	10
…when it is not part of my daily tasks	0	1	2	3	4	5	6	7	8	9	10
…when I am busy (pressure from work, too much work at home, family responsibilities)	0	1	2	3	4	5	6	7	8	9	10
…when I have to exercise alone	0	1	2	3	4	5	6	7	8	9	10
…if I don't reach my exercise goals	0	1	2	3	4	5	6	7	8	9	10
…after recovering from illness or after vacation	0	1	2	3	4	5	6	7	8	9	10
… when I feel physical discomfort when I exercise	0	1	2	3	4	5	6	7	8	9	10

Where do I score high?

How can I act differently in the future?

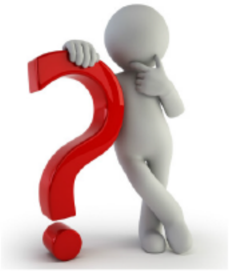

Discuss in the group:

1. When is it particularly difficult to exercise?
2. Suggestions to make it easier in the future?

Facilitator tips:

1. Highlight and link participants with similar concerns and solutions in the group
2. Summarize similar issues to help participants see topics in a new way by hearing and considering new ideas and perspectives from others having the same challenges
