# Supplementary material for: Group-based, person-centered diabetes self-management education: healthcare professionals’ implementation of new approaches
Source: BMC Health Serv Res. 2019 Jun 11;19:368. doi: 10.1186/s12913-019-4183-1 (PMC6558764; doi:10.1186/s12913-019-4183-1)
Supplement: Supplementary file 3 — Tool to facilitate reflection and dialog about eating habits. (PDF 114 kb) [file 12913_2019_4183_MOESM3_ESM.pdf]

**Supplementary file 3.** Tool to facilitate reflection and dialog about eating habits

## MY EATING HABITS

Eating is normally more influenced by the principles of pleasure and social interaction. Please circle the scale below. How strongly do you agree with the following statements?

| My eating habits depend on… | Strongly disagree | | | | | | Strongly agree |
|---|---|---|---|---|---|---|---|
| …my emotions (boredom, loneliness, sadness, reward, guilt, anger, happiness) | 0 | 1 | 2 | 3 | 4 | 5 | 6 |
| …my physical discomfort (stress, tiredness, exhaustion, pain) | 0 | 1 | 2 | 3 | 4 | 5 | 6 |
| …my daily routines (TV, reading, late at night) | 0 | 1 | 2 | 3 | 4 | 5 | 6 |
| …availability or social life (friends, family, weekends, holiday) | 0 | 1 | 2 | 3 | 4 | 5 | 6 |

**Try to describe the certain events that trigger your unhelpful eating habits?**
(Emotions, physical discomfort, daily routines, availability or social life?)



**How can you manage your eating habits differently?**

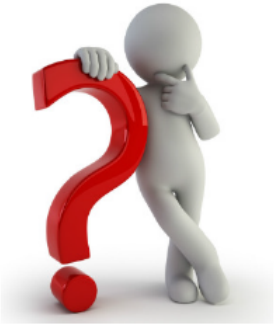

**Discuss in the group:**

1. Triggers to food?
2. Alternative activities to food?

**Facilitator tips:**

1. Highlight and link participants with similar concerns and solutions in the group
2. Summarize similar issues to help participants see topics in a new way by hearing and considering new ideas and perspectives from others having the same challenges
