# Supplementary material for: Group-based, person-centered diabetes self-management education: healthcare professionals’ implementation of new approaches
Source: BMC Health Serv Res. 2019 Jun 11;19:368. doi: 10.1186/s12913-019-4183-1 (PMC6558764; doi:10.1186/s12913-019-4183-1)
Supplement: Supplementary file 4 — Tool to assess readiness to change and facilitation techniques. (PDF 443 kb) [file 12913_2019_4183_MOESM4_ESM.pdf]

Supplementary file 4. Tool to assess readiness to change and facilitation techniques

READY TO CHANGE?

NO	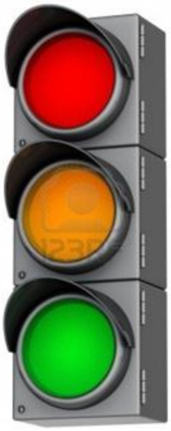	Not ready to change	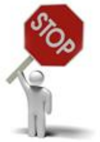
MAYBE		Pros and cons	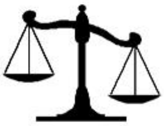
YES		Ready to change	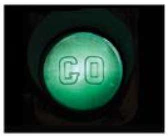

READY TO CHANGE?

Choose one issue in life with diabetes that you have considered changing: ______________________

Are you ready to change?

- ❑ NO
- ❑ MAYBE
- ❑ YES

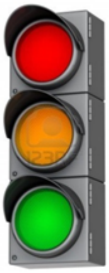

Why is it important to you?

____

____

____

____

Supplementary file 4. Tool to assess readiness to change and facilitation techniques

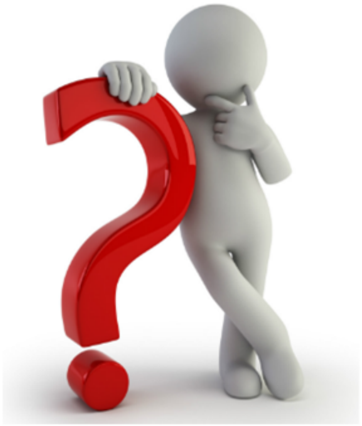

Discuss in the group what you have considered to change in your daily life with diabetes?

Facilitator tips:
Be aware that "green light" assessment can be disguised as "red or yellow light"

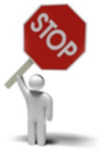

NOT READY TO CHANGE

FACILITATORS	BARRIERS
Keep the conversation going to maintain the relationship:<br>✓ Explore why they are not ready to change<br>✓ Acknowledge and normalize the challenges of living with diabetes<br>✓ Use a non-judgmental approach<br>✓ Ask open-ended questions (don't tell them what to do)<br>✓ Ask for permission before giving advice<br>✓ Explore resistance actively, rather than confronting the person judgmentally. Try to be curious in negative statements<br><br>Initiate a dialogue to clarify personal values	Give advice and suggest solutions to the challenges the participants might have<br><br>Giving advice without permission<br>➢ Invites to resistance<br><br>Urging more willpower<br><br>Threatening bad outcomes (diabetes complications)<br><br>Defensiveness/confrontation/ arguing for change<br>➢ Can cause defensiveness and resistance<br>➢ Shame and blame can destroy motivation<br>➢ Can establish a negative relationship

Supplementary file 4. Tool to assess readiness to change and facilitation techniques

PROS & CONS

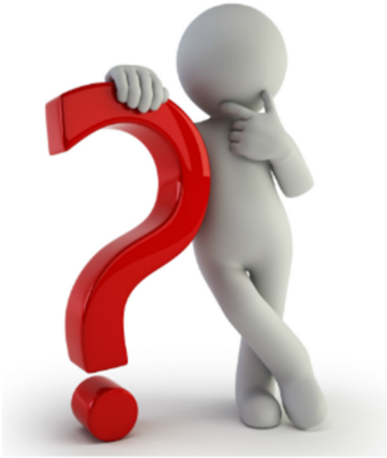

Ask the group to consider and present pros- and cons of changing or staying the same (divide the group into subgroups and note down their answers)

Summarize

Ask each group participant to mention one item on the chart, which they noticed and found meaningful

PROS & CONS

	PROS	CONS
Staying the same		
Changing		

Supplementary file 4. Tool to assess readiness to change and facilitation techniques

READY TO CHANGE

	ASK	LISTEN (includes statements of acknowledgement)	REFLECT	SUMMARIZING STATEMENTS
STRATEGY	Open-ended questions to:<br>1) Identify needs, preferences, and values<br>2) Elicit what the group knows and ask for permission before presenting information	Highlight and acknowledge positive actions<br>Demonstrate an understanding of key messages from the group<br>Allow for silence	Acknowledge and mirror key messages back to the whole group<br>Ask open-ended questions as a way to engage the group to reflect and find common issues	*Summarizing:*<br>Brings different statements from the group and end with an open question inviting and adding multiple views<br>*Connecting:*<br>Connect earlier statements and highlight shared perspectives<br>*Change subject:*<br>Summarize the subject and change to a new and more productive subject
AIM	Gives a feeling of ownership and engagement<br>Not exclusively attached to the curriculum	Builds up motivation and supports self-efficacy<br>Respect and accept participants as they are unconditionally	Gives an opportunity for participants to elaborate and expand on their perspectives and thoughts	Enables participants to take a step back and see the overall picture<br>Statements about change applies to everyone in the group
EXAMPLE/ TECHNIQUE	What's living with diabetes been like for you?<br>What significance does diabetes have in my daily life?	Look at the whole group when someone talks. It invites others into the conversations and supports a group conversation	What I'm hearing you are saying is…<br>I'm hearing that it's important to you…<br>Have I understood this correctly when you are saying…<br>Who else?<br>What else?	Who else in the group can recognize what XX just told?<br>What have you done when you were in a similar situation?<br>I can see that many of you are nodding. I think that many of you can recognize what XX just told- is that true?
